# Complete genome sequences of five *Escherichia coli* strains with probiotic attributes

**DOI:** 10.1128/MRA.00363-23

**Published:** 2023-08-07

**Authors:** Pavla Fedrová, Matěj Hrala, Nikola Tom, Lenka Micenková, Andréa M. A. Nascimento, Juraj Bosák, David Šmajs

**Affiliations:** 1 Department of Biology, Faculty of Medicine, Masaryk University, Brno, Czech Republic; 2 Department of Experimental Biology, Faculty of Science, Masaryk University, Brno, Czech Republic; 3 Departamento de Biologia Geral, Instituto de Ciências Biológicas, Universidade Federal de Minas Gerais, Belo Horizonte, Brazil; Wellesley College, Wellesley, Massachusetts, USA

**Keywords:** *Escherichia*, *Escherichia coli*, probiotics

## Abstract

The complete genome sequences of five *Escherichia coli* strains with probiotic attributes were determined, including strain A0 34/86, a component of the probiotic product Colinfant New Born, and strains H22, 582, B771, and B1172 with published probiotic potential. The size of sequenced genomes ranged from 5,092 to 5,408 kb.

## ANNOUNCEMENT

*Escherichia coli* (*E. coli*) has significant importance for human health (1). Until now, only a few strains with beneficial features have been applied in human medicine as probiotics (2). Here, *E. coli* strain A0 34/86, a component of commercially available probiotic preparation approved in the Czech and Slovak Republics (3), was sequenced. In addition, *E. coli* H22, 582, B771, and B1172 strains with previously published probiotic attributes (4, 5) were sequenced. *E. coli* strains were previously isolated from fecal samples in accordance with the Declaration of Helsinki and are part of our laboratory collection.

*E. coli* strains from our laboratory stocks were cultivated overnight in tryptone-yeast broth at 37°C and 200 rpm. The total DNA was isolated using phenol-chloroform extraction (5 × 10^9^ cells) (6) and used for Illumina sequencing (150 bp-long, paired-end reads). Library preparation (NEBNext DNA Library Prep Kit, NEB), sequencing (HiSeq4000), and read processing (i.e., removal of adapters and low-quality reads - Q_phred_ < 5, *N* > 10%) were performed at the Novogene facility (China). In addition, total DNA isolated from 2 × 10^9^ cells with MagAttract Microbial DNA Kit (Qiagen) was used for long-read sequencing with the MinION platform (Oxford Nanopore Technologies, ONT). Unsheared and non-size-selected DNA was used for the preparation of libraries (SQK-LSK108 ligation kit [ONT] and EXP-NBD103 barcoding kit [ONT]) that were sequenced on the SpotON flow cell (R9). Read processing was performed using MinKNOW v19.06.07 (ONT, fast base-calling Q > 7, adapter trimming) and the online portal Epi2me (ONT, barcode trimming). Reads from both sequencing platforms were used for *de novo* assembly. Genomes were assembled using Unicycler v0.4.8 with default parameters (7). If necessary, the assembly was completed using Flye v2.6 (8), SPAdes v3.13.1 (9, 10), Minimap v2.1 (11), and SAMtools v1.9 (12). Finally, all genomes were manually inspected using Lasergene software v7.1.0 (DNASTAR), and two regions with low coverage were verified with Sanger sequencing (CP120567: 5,097,983–5,098,209 and CP120559: 93,482–94,709). Genome circularization was performed during assembly using Unicycler software (v0.4.8) with default parameters. Genomes were annotated using the Prokaryotic genome annotation pipeline (v6, NCBI). Additionally, complete genomes were used for phylogroup and serotype classifications using online tools ClermonTyping (http://clermontyping.iame-research.center/ [13]) and SerorypeFinder v2.0 (https://cge.food.dtu.dk/services/SerotypeFinder/ [14]) with default parameters.

Complete genome sequences were obtained for all five *E. coli* strains, with an average coverage between 270× and 621×. The genome size ranged from 5,092 to 5,408 kbp. The probiotic A0 34/86 strain contained a single circular chromosome with no plasmids, while up to seven extrachromosomal plasmid molecules were found in the other strains. Detailed information about whole-genome sequencing parameters and genome characteristics for individual *E. coli* strains is shown in Table 1.

**TABLE 1:** Whole-genome characteristics of the sequenced *E. coli* probiotic strains

	*E. coli* strain				
	A0 34/86	H22	B771	B1172	582
No. of Illumina reads	5,371,754	6,360,832	5,694,844	12,446,381	6,665,029
No. of Nanopore reads (N50; bp)	54,557 (8,094)	72,245 (4,997)	106,406 (4,380)	73,477 (2,754)	49,426 (8,559)
Genome coverage	324×	368×	270×	621×	395×
Genome GC content (%)	50.46	50.51	50.78	50.65	50.81
Genome size (bp)	5,098,211	5,161,491	5,341,634	5,408,598	5,092,660
Chromosome size (bp)	5,098,211	5,029,969	5,103,692	5,147,947	4,870,922
Plasmids and size (bp)^*a*^	-	122,286; 5,159; 4,077	128,330; 95,480; 9,494; 2,328; 2,310	153,456; 77,638; 11,599; 6,988; 5,164; 4,241; 1,565	124,850; 6,888
No. of predicted genes	4,938	5,061	5,247	5,281	4,913
No. of RNA genes	117	117	114	116	124
No. of pseudogenes	166	197	304	243	225
*E. coli* serotype	O83:H31	O88:H14	O11:H4	O2/O50:H6	O88:H4
*E. coli* phylogroup	B2	B2	A	B2	B2
BioSample number	SAMN33748624	SAMN33748625	SAMN33748626	SAMN33748627	SAMN33748628

^*a*^ Plasmids represent circular molecules obtained from *in silico* assembly and were not experimentally determined.

## Data Availability

The complete genomes were deposited in GenBank: strain A0 34/86 (chromosome CP120567); strain H22 (chromosome CP120563 and plasmids CP120564, CP120565, CP120566); strain B771 (chromosome CP120557 and plasmids CP120558, CP120559, CP120560, CP120561, CP120562); strain B1172 (chromosome CP120549, and plasmids CP120550, CP120551, CP120552, CP120553, CP120554, CP120555, CP120556); 582 (chromosome CP120568, and plasmids CP120569, CP120570). Corresponding SRA data were deposited under numbers SRS17445630, SRS17446543, SRS17446542, SRS17511476, and SRS17445631.

## References

[1] Tenaillon O , Skurnik D , Picard B , Denamur E . 2010. The population genetics of commensal Escherichia coli. Nat Rev Microbiol 8:207–217. doi:10.1038/nrmicro2298 20157339

[2] Wassenaar TM . 2016. Insights from 100 years of research with probiotic E. coli. Eur J Microbiol Immunol 6:147–161. doi:10.1556/1886.2016.00029 PMC5063008 27766164

[3] Micenková L , Bosák J , Smatana S , Novotný A , Budinská E , Šmajs D . 2020. Administration of the probiotic Escherichia coli strain A0 34/86 resulted in a stable colonization of the human intestine during the first year of life. Probiotics Antimicrob Proteins 12:343–350. doi:10.1007/s12602-019-09548-3 31069717

[4] Cursino L , Šmajs D , Šmarda J , Nardi RMD , Nicoli JR , Chartone-Souza E , Nascimento AMA . 2006. Exoproducts of the Escherichia coli strain H22 inhibiting some enteric pathogens both in vitro and in vivo. J Appl Microbiol 100:821–829. doi:10.1111/j.1365-2672.2006.02834.x 16553738

[5] Hrala M , Bosák J , Micenková L , Křenová J , Lexa M , Pirková V , Tomáštíková Z , Koláčková I , Šmajs D . 2021. Escherichia coli strains producing selected bacteriocins inhibit porcine enterotoxigenic Escherichia coli (ETEC) under both in vitro and in vivo conditions. Appl Environ Microbiol 87:e0312120. doi:10.1128/AEM.03121-20 33962981 PMC8231719

[6] Butler JM . 2012. Chapter 2, DNA extraction methods, p 29–47. In Butler JM (ed), Advanced topics in forensic DNA typing: methodology. Academic Press. doi:10.1016/B978-0-12-374513-2.00002-6

[7] Wick RR , Judd LM , Gorrie CL , Holt KE . 2017. Unicycler: resolving bacterial genome assemblies from short and long sequencing reads. PLoS Comput Biol 13:e1005595. doi:10.1371/journal.pcbi.1005595 28594827 PMC5481147

[8] Kolmogorov M , Yuan J , Lin Y , Pevzner PA . 2019. Assembly of long, error-prone reads using repeat graphs. Nat Biotechnol 37:540–546. doi:10.1038/s41587-019-0072-8 30936562

[9] Antipov D , Korobeynikov A , McLean JS , Pevzner PA . 2016. HybridSPAdes: an algorithm for hybrid assembly of short and long reads. Bioinformatics 32:1009–1015. doi:10.1093/bioinformatics/btv688 26589280 PMC4907386

[10] Prjibelski A , Antipov D , Meleshko D , Lapidus A , Korobeynikov A . 2020. Using SPAdes de novo assembler. Curr Protoc Bioinformatics 70:e102. doi:10.1002/cpbi.102 32559359

[11] Li H . 2018. Minimap2: pairwise alignment for nucleotide sequences. Bioinformatics 34:3094–3100. doi:10.1093/bioinformatics/bty191 29750242 PMC6137996

[12] Danecek P , Bonfield JK , Liddle J , Marshall J , Ohan V , Pollard MO , Whitwham A , Keane T , McCarthy SA , Davies RM , Li H . 2021. Twelve years of SAMtools and BCFtools. Gigascience 10:giab008. doi:10.1093/gigascience/giab008 33590861 PMC7931819

[13] Beghain J , Bridier-Nahmias A , Le Nagard H , Denamur E , Clermont O . 2018. Clermontyping: an easy-to-use and accurate in silico method for Escherichia genus strain phylotyping. Microb Genom 4:e000192. doi:10.1099/mgen.0.000192 29916797 PMC6113867

[14] Joensen KG , Tetzschner AMM , Iguchi A , Aarestrup FM , Scheutz F . 2015. Rapid and easy in silico serotyping of Escherichia coli isolates by use of whole-genome sequencing data. J Clin Microbiol 53:2410–2426. doi:10.1128/JCM.00008-15 25972421 PMC4508402

